# Recurrent Kimura disease in an African American woman with concordant ultrasound imaging: A case report and literature review

**DOI:** 10.1016/j.jdcr.2025.04.019

**Published:** 2025-04-26

**Authors:** Janet Choi, Jonathan Sterman, Hatice Zengin, Bijal Amin, Benedict Wu

**Affiliations:** a Division of Dermatology, Albert Einstein College of Medicine/Montefiore Medical Center, Bronx, New York; b Department of Radiology, Albert Einstein College of Medicine/Montefiore Medical Center, Bronx, New York; c Department of Pathology, Albert Einstein College of Medicine/Montefiore Medical Center, Bronx, New York

**Keywords:** Kimura disease, reactive lymphadenopathy, subcutaneous mass

## Introduction

Kimura disease (KD) is a rare, benign condition characterized by hyperplastic lymphoid granuloma in the head and neck region, peripheral eosinophilia, and elevated serum immunoglobulin E levels. KD predominately occurs in young Asian men, but has been reported in 1 African American (AA) man.^1^ Diagnosing KD may be challenging as it can present similar to other lymphoid conditions such as Hodgkin’s disease, Langerhans cell histiocytosis, and reactive lymphadenopathy related to infections.^2^ Ultrasound can be diagnostic and assist with determination of nodule size and lymph node involvement. Herein, we present a case of KD in an AA woman with supportive ultrasound findings.

## Case report

**Fig 1:**
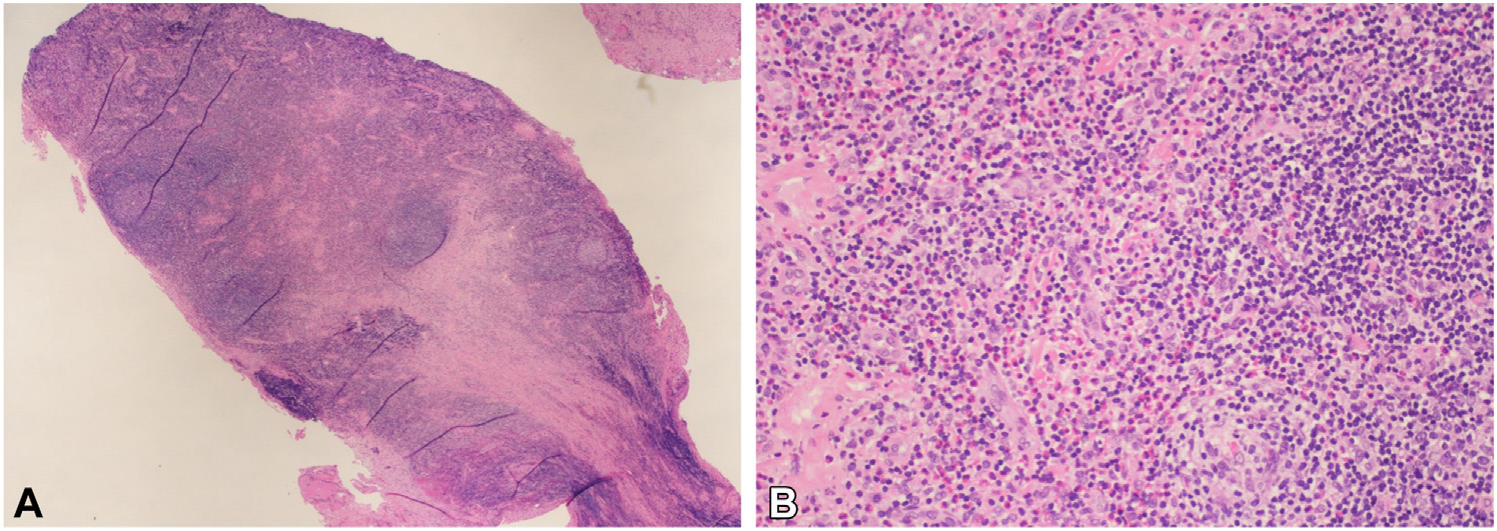
Histopathology of Kimura disease. Hematoxylin and eosin (H&E) staining. **A,** H&E, 2×: A section shows dense lymphoid aggregates. **B,** H&E, 20×: The lymphoid infiltrate is accompanied by numerous eosinophils.

A 39-year-old AA woman with a history of morbid obesity and hypertension presented with a 3-cm mobile nodule on her left cheek, associated with intermittent left facial swelling. A 6-mm punch biopsy revealed blood vessels and fibromuscular tissue containing lymphoid aggregates associated with many eosinophils, suggesting KD (Fig 1, *A* and *B*).

**Fig 2:**
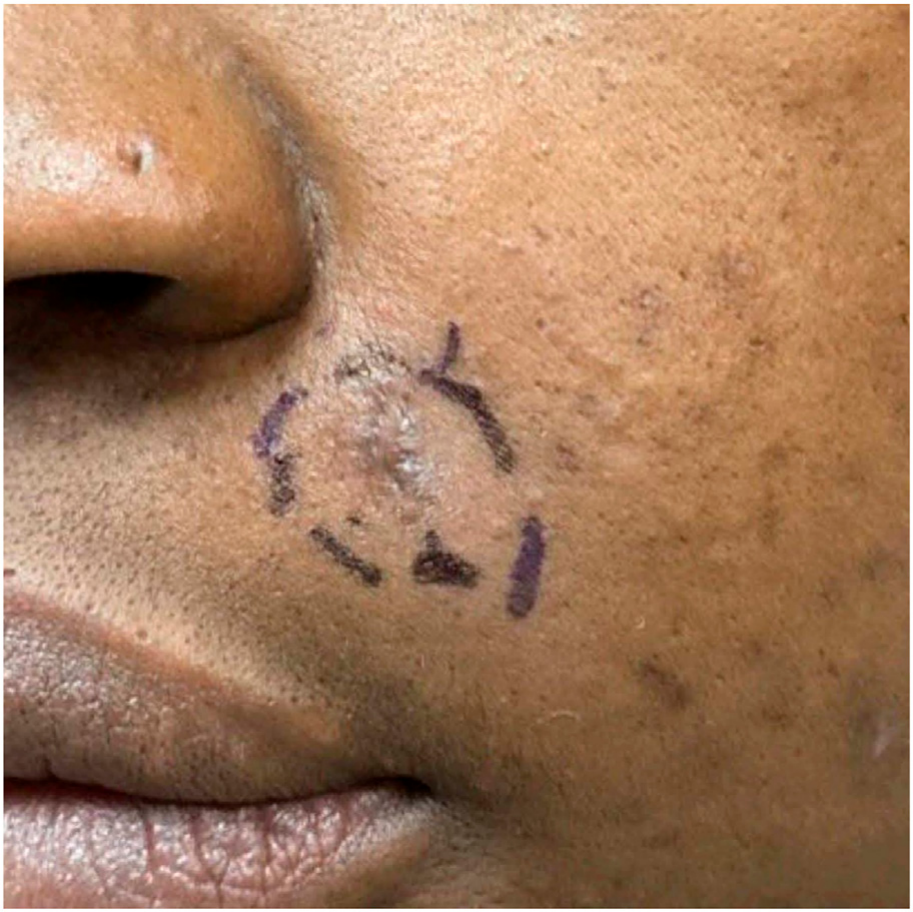
Kimura disease: Clinical image of a nodule on the left cheek after the punch biopsy but before the excisional biopsy.

Laboratory testing revealed elevated immunoglobulin E levels but normal eosinophil counts. 4 months later, the nodule reappeared at the exact location (Fig 2), and the excisional biopsy showed similar histopathologic findings.

**Fig 3:**
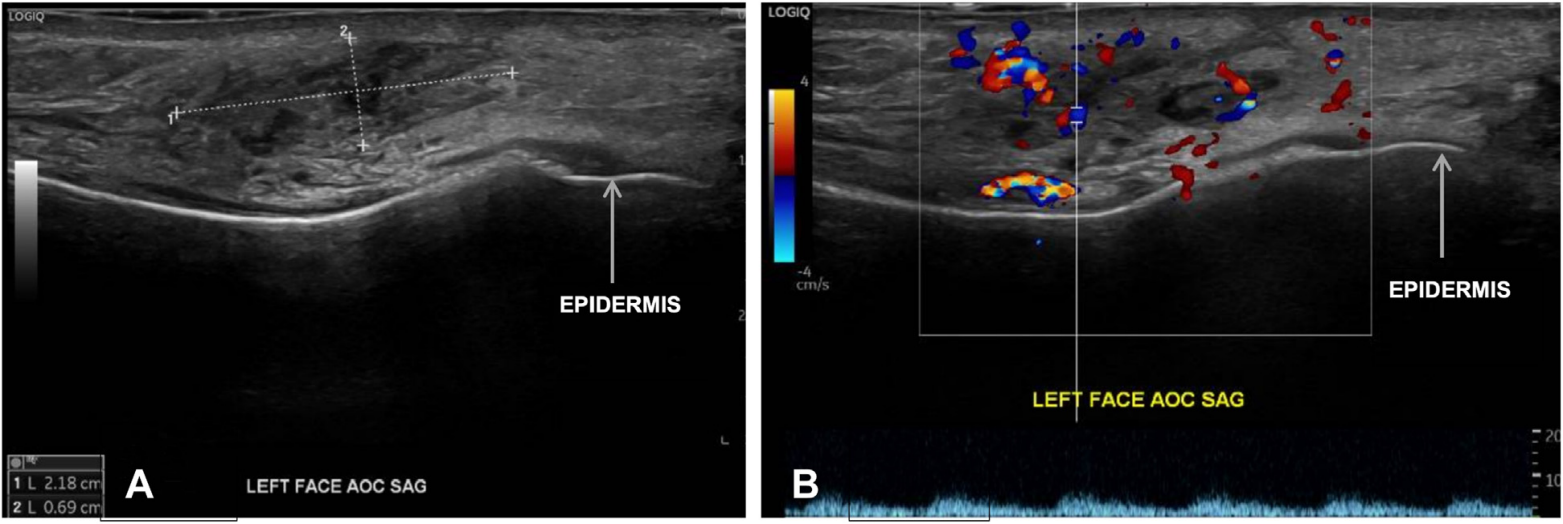
Kimura disease ultrasound. **A,** Heterogeneous 2.2 × 0.7 cm area of subcutaneous soft tissue thickening on the left face. **B,** Color Doppler imaging of left face identifying areas of hypervascularity and multiple, normal-sized lymph nodes. *AOC*, Area of concern; *SAG*, sagittal view.

Ultrasound of the affected area demonstrated heterogeneous soft tissue thickening (Fig 3, *A*) and regions of hypervascularity containing multiple, normal-sized lymph nodes (Fig 3, *B*), aligning with reported sonographic features of KD.^3^

## Discussion

KD is a chronic inflammatory disorder typically manifesting as a painless subcutaneous nodule or soft tissue swelling in the head and neck region, often invading nearby lymph nodes or parotid glands. Ultrasound is a valuable initial diagnostic resource, commonly revealing hypoechoic, round nodes with partially defined margins, and associated regional lymphadenopathy with preserved fatty hilum.^3^^,^^4^ Color Doppler sonograms often show hilar vascularity. In contrast, malignant nodes frequently show capsular vascularity and loss of hilar architecture.^3^ Confirmation of KD is often done via histopathologic evaluation. Treatment options for KD include oral corticosteroids, antihistamines, biologics, cytotoxic therapy, excision, and radiation, although recurrence rates are high at 56.3%.^1^^,^^5^ Recurrence risks include systemic diseases such as hypertension and obesity, both of which were seen in our patient.^6^

We reviewed all reported cases of adult KD in the head and neck with descriptive sonographic findings over the past 5 years (Table I).

**Table I:** Reported cases of adult Kimura disease with descriptive ultrasound findings

Author last name, y	Age/sex	Race/ethnicity	Peripheral eosinophilia	IgE level	Ultrasound findings						Diagnostic modalities	Treatment	Treatment response
					Location	Margin	Echogenicity	Internal vascularity on color Doppler	Lymph nodes with maintained fatty hilum	Adjacent lymphadenopathy			
Maehara, 2019^7^	56/M	Asian/Japanese	Present	Elevated at 14,834 IU/mL (N: N/A)	Left parotid region	N/A	Reticular pattern of hypoechoic areas	Present	N/A	Present	Imaging,∗ histopathology	Systemic medication†	Reduction in size with normalization of peripheral eosinophilia but persistently elevated IgE levels
Prayuenyong, 2022^8^	22/M	Asian/Thai	Present	N/A	Left parotid and periparotid regions	Well defined	Hypoechoic	Present	N/A	Present	Imaging,‡ histopathology	Superficial parotidectomy	Resolution without recurrence
Sangwan, 2020^4^	22/M	N/A	Present	N/A	Right parotid region	Ill-defined	Predominantly hyperechoic with hypoechoic areas within	Present	Present	Present	Imaging,∗ histopathology	Patient is planned for surgical excision	N/A
Shivakumar, 2021^9^	35/M	N/A	Present	N/A	Right parotid region	N/A	Heterogenous gland with altered echotexture	N/A	Present	Present	Imaging,‡ histopathology	Systemic medication†	Mild reduction in size
Yang, 2022^5^	57/M	Asian/Chinese	Present	Elevated at >2000 IU/mL (N: <100 IU/mL)	Bilateral anterior and posterior ears	N/A	Heterogeneously hypoechoic-isoechoic	N/A	N/A	Present	Histopathology	Systemic medication§	Reduction in size with normalization of peripheral eosinophilia but persistently elevated IgE. Follow-up is ongoing.
Yorita, 2023^10^	19/F	N/A	Present	Elevated at 424.5 IU/mL (N: <170 IU/mL)	Right parotid region	N/A	Homogenously hypoechoic	N/A	N/A	N/A‖	Imaging,∗^,^‡ histopathology	N/A	N/A
Present case	39/F	African American/non-Hispanic	Absent	Elevated at 124.0 IU/mL (N: <100 IU/mL)	Left cheek	Ill-defined	Predominately hypoechoic with central hyperechoic areas within	Present	Present	Absent	Histopathology	Excision	Recurrence after first punch biopsy. Lost to follow-up after excision.

*F*, Female; *IgE*, immunoglobulin E; *M*, male; *N*, normal; *N/A*, not available.

∗ MRI.

† Oral corticosteroid therapy.

‡ CT.

§ First with corticosteroid therapy (prednisone), then biologic therapy (omalizumab then dupilumab).

ǁ Lymphadenopathy observed on physical examination but not mentioned for ultrasound.

Four of 6 identified cases had diagnostic ultrasounds and biopsies performed. Reported ultrasound features included hypoechoic lesions (*n* = 5), peripheral lymphadenopathy (*n* = 4), internal vascularity (*n* = 3), and preserved fatty hilum (*n* = 2). Our patient demonstrated all these findings except for peripheral lymphadenopathy, possibly due to a more localized degree of tissue involvement. Additional imaging techniques including magnetic resonance imaging (*n* = 3) and computed tomography (*n* = 3) were used to determine the extent of disease.

We present this case of recurrent KD in an AA woman to enrich the limited reports of KD in non-Asian patients and to show ultrasound imaging as a potentially valuable tool in diagnosis and disease surveillance. Of note, as KD may mimic features of Hodgkin’s disease, hematologic malignancies should be prioritized in the differential diagnosis in older patients.^6^ However, in cases where malignancy is less likely and histopathologic findings are equivocal, ultrasound may be a practical, noninvasive diagnostic method.

### Data sharing and data availability

Data sharing is not applicable to this article as no datasets were generated or analyzed during the present study.

## Conflicts of interest

None disclosed.
